# HCMV trimer- and pentamer-specific antibodies synergize for virus neutralization but do not correlate with congenital transmission

**DOI:** 10.1073/pnas.1814835116

**Published:** 2019-02-07

**Authors:** Adam L. Vanarsdall, Andrea L. Chin, Jing Liu, Theodore S. Jardetzky, James O. Mudd, Susan L. Orloff, Daniel Streblow, Marisa M. Mussi-Pinhata, Aparecida Y. Yamamoto, Geraldo Duarte, William J. Britt, David C. Johnson

**Affiliations:** ^a^Department of Molecular Microbiology & Immunology, Oregon Health & Science University, Portland, OR 97239;; ^b^Department of Structural Biology, Stanford University, Stanford, CA 94305;; ^c^Knight Cardiovascular Institute, Oregon Health & Science University, Portland, OR 97239;; ^d^Department of Surgery, Oregon Health & Science University, Portland, OR 97239;; ^e^Vaccine and Gene Therapy Institute, Oregon Health & Science University, Beaverton, OR 97006;; ^f^Department of Pediatrics, Ribeirão Preto Medical School, University of São Paulo, São Paulo 14049, Brazil;; ^g^Department of Gynecology and Obstetrics, Ribeirão Preto Medical School, University of São Paulo, São Paulo 14049, Brazil;; ^h^Department of Pediatrics, Microbiology & Neurobiology, University of Alabama, Birmingham, AL 35233

**Keywords:** congenital, neutralizing antibodies, transplant, cell-to-cell spread, vaccines

## Abstract

Human cytomegalovirus (HCMV) causes substantial disease in transplant patients and harms the development of the nervous system in babies infected in utero. Thus, there is a major focus on developing safe and effective HCMV vaccines. Evidence has been presented that a major target of neutralizing antibodies (NAbs) is the HCMV pentamer glycoprotein gH/gL/UL128-131. In some studies, most of the NAbs in animal or human sera were found to recognize the pentamer, which mediates HCMV entry into endothelial and epithelial cells. It was also reported that pentamer-specific antibodies correlate with protection against transmission from mothers to babies. One problem with the studies on pentamer-specific NAbs to date has been that the studies did not compare the pentamer to the other major form of gH/gL, the gH/gL/gO trimer, which is essential for entry into all cell types. Here, we demonstrate that both trimer and pentamer NAbs are frequently found in human transplant patients’ and pregnant mothers’ sera. Depletion of human sera with trimer caused reductions in NAbs similar to that observed following depletion with the pentamer. The trimer- and pentamer-specific antibodies acted in a synergistic fashion to neutralize HCMV and also to prevent virus cell-to-cell spread. Importantly, there was no correlation between the titers of trimer- and pentamer-specific NAbs and transmission of HCMV from mothers to babies. Therefore, both the trimer and pentamer are important targets of NAbs. Nevertheless, these antibodies do not protect against transmission of HCMV from mothers to babies.

## Significance

Human cytomegalovirus (HCMV) causes severe morbidity and mortality in immunocompromised patients and is the most commonly transmitted virus that causes developmental defects in the fetus. Currently, there is no licensed HCMV vaccine available, and prior efforts using attenuated viruses and subunit vaccines were not successful. Recently, there has been intense interest in the HCMV pentamer glycoprotein as a component of vaccines. Here, we show that transplant patients’ and pregnant mothers’ sera contain neutralizing antibodies specific for the pentamer and also for a second HCMV glycoprotein, the trimer, which is essential for HCMV entry into cells. Trimer- and pentamer-specific antibodies acted synergistically to neutralize virus and block cell–cell spread. These observations will have major implications for the future of HCMV vaccine development.

Human cytomegalovirus (HCMV) is the most common infection acquired by developing infants, affecting ∼0.5% of newborn babies (1–4). In the United States, HCMV accounts for 25% of children with sensorineural hearing loss (5). Some studies have indicated that antibodies (Abs) can prevent or reduce HCMV spread from mother to infants in utero. Specifically, women with primary HCMV infection that occurred during or shortly before pregnancy were given monthly infusions of HCMV hyperimmune IgG that protected ∼60% of the babies, although these findings were not confirmed in a more rigorous trial of the same preparation of hyperimmune IgG (6–8). There was also evidence that certain HCMV neutralizing Abs (NAbs) correlate with protection against transmission of HCMV from mother to child (5). These NAbs may be especially important given that Abs appear earlier than T cell responses following primary infection (9, 10). However, a different view of the role of Abs in HCMV congenital disease has been reported. Mothers with high levels of anti-HCMV NAbs transmit virus to developing fetuses with frequencies similar to those of seronegative mothers (4). Brazilian women exposed to HCMV infection at young ages have high rates of seroprevalence (97%) (11). In this population, most of the HCMV-infected infants were born to women who were seropositive before pregnancy with high titers of anti-HCMV NAbs (12). Thus, some studies support the conclusion that Abs protect against transmission to babies, while others argue that this is not the case.

HCMV infects a large number of cell types in vivo, including endothelial and epithelial cells, fibroblasts, and leukocytes (10, 13). Monocytes and macrophages and leukocytes carrying HCMV are thought to interact with endothelial cells that act as portals for HCMV from the blood into solid tissues where hepatocytes, epithelial cells, and fibroblasts are infected. In contrast, laboratory strains of HCMV, including AD169 and Towne, that have been adapted to growth in fibroblasts do not infect monocytes and macrophages or epithelial and endothelial cells well due to the lack of three genes: UL128, UL130, and UL131 (10, 13). The UL128-131 genes encode three proteins that assemble onto HCMV glycoprotein gH/gL (14) to produce the gH/gL/UL128-131 complex, also known as the pentamer. The pentamer mediates virus entry into endothelial and epithelial cells and monocytes and macrophages (15, 16).

Numerous studies have demonstrated that pentamer-specific Abs are highly neutralizing and important in Ab-mediated control of HCMV. For example, monoclonal Abs (MAbs) from humans that neutralized HCMV were isolated, and a large fraction of these recognized the pentamer (17). A major fraction of NAb responses in human hyperimmune globulin was specific for the pentamer and did not recognize gH/gL or gB, the other major HCMV glycoprotein involved in virus entry (18). Potent neutralizing MAbs elicited in rabbits immunized with a HCMV vaccine candidate preferentially recognized the pentamer and not gB (19). A soluble form of the pentamer adsorbed 76% of the NAbs in human hyperimmune globulin, whereas soluble gH/gL was much less effective (20). Other studies involving immunization of experimental animals confirmed that the pentamer is an important target of NAbs (21, 22). Further, there was a description of a correlation between pentamer-specific Abs and disease outcome in these studies. Characterization of sera from mothers who became infected with HCMV during or just before pregnancy revealed that pentamer-specific Abs were higher in mothers who did not transmit virus compared with mothers who transmitted HCMV to their babies, yet Abs specific for gB were not different (5). Moreover, sera from nontransmitters contained higher levels of Abs that could block the binding of pentamer-specific neutralizing MAbs. That said, the quantities of pentamer-specific IgG in transmitters versus nontransmitters were not largely different and were observed only in the first 30 d following primary infection. However, these and other studies and reviews have stressed the importance of pentamer-specific Abs in the design of HCMV vaccines (23–30).

Comparisons between pentamer-specific Abs and Abs specific for gH/gL or gB pose several problems. There is evidence that gH/gL itself, without the UL128-131 or other viral proteins, may not be present in virus particles at substantial levels (31, 32). The major form of gH/gL in virions is a covalent complex of gH/gL and gO known as the trimer (23, 24, 33). We recently described a third gH/gL complex with gB, and gB-gH/gL makes up a significant fraction of gH/gL in the virion envelope (32). The gH/gL/gO trimer is essential for virus entry into all cell types (23), but it is not yet clear whether the gB-gH/gL complex is essential (32). Given that the trimer is essential in all cell types, it would seem that Abs that recognize the trimer should also neutralize HCMV and perhaps block transmission to babies, but this has not been tested. HCMV gB is thought to be triggered to produce membrane fusion following gB activation by either the trimer or pentamer or both (13, 25). Thus, it would also seem that gB-specific Abs should also be neutralizing. However, prior comparisons of the pentamer to gB Abs have involved a soluble form of HCMV gB which, like the herpes simplex virus and Epstein–Barr virus gB molecules, is in the postfusion form (26–29), unlike full-length HSV gB, which is apparently in a distinct prefusion conformation (30). Consequently, it is likely that there are gB-specific NAbs in human sera that recognize prefusion gB epitopes.

## Results

### Sera from HCMV-Positive Human Transplant Patients Contain both Trimer- and Pentamer-Specific NAbs.

**Fig. 1.**
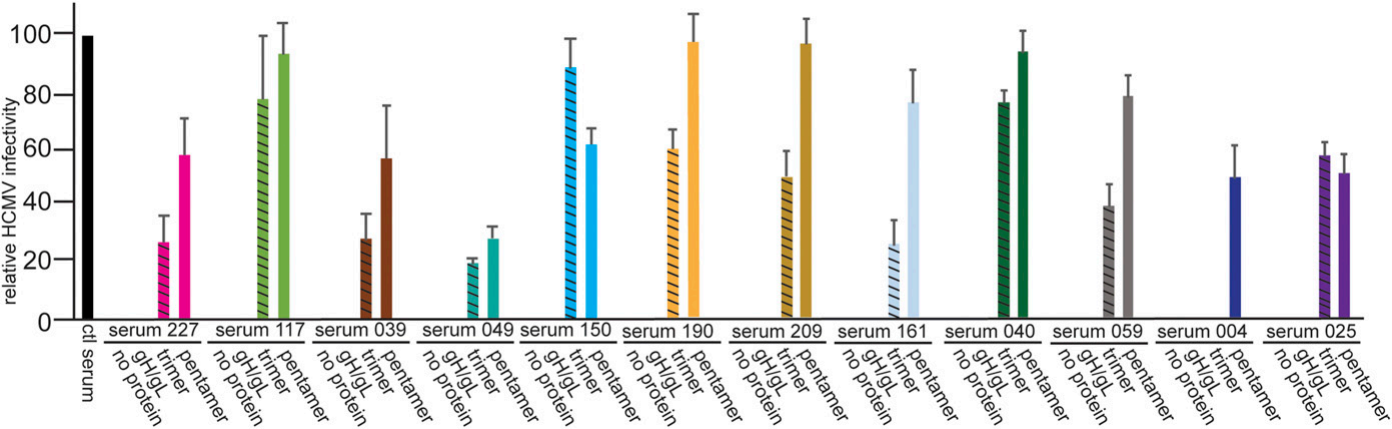
Neutralizing capacity of human serum from transplant patients following depletion with gH/gL, trimer, or pentamer. Human sera collected following heart transplant were diluted to titers that resulted in 100% neutralization of HCMV infection of APRE-19 cells (NT_100_). The diluted sera were then incubated with either no protein or with 1 μg of soluble gH/gL, trimer, or pentamer and then tested for the ability to neutralize HCMV (BAD*r*UL131) infection of APRE-19 epithelial cells. The relative infectivity of HCMV after incubation with sera was determined by counting GFP+ (infected) cells after 24 h of infection and compared with the number of infected cells following incubation of HCMV with a seronegative donor serum (ctl serum).

We characterized sera from a panel of human heart transplant patients. Patient demographics are listed in *SI Appendix*, Table S1. Most of the patients received prophylaxis therapy to prevent HCMV disease, but six of the patients experienced substantial HCMV disease posttransplant and were treated for viremia. HCMV neutralizing titers [i.e., the dilution of sera resulting in 50% (NT_50_) or 100% neutralization (NT_100_)] were determined in full dilution curves involving twofold dilutions followed by incubation of sera with HCMV BAD*r*UL131 followed by infection of APRE-19 epithelial cells. There is not sufficient space to show each of these neutralization curves, but *SI Appendix*, Table S2 shows NT_50_ and NT_100_ values. NT_100_ dilutions were used in all of the antibody depletion experiments that follow. Diluted sera were incubated with 1 μg of soluble gH/gL, trimer, or pentamer complexes that were purified as described previously (34). Dose–response experiments established that 1 μg of each of these soluble proteins was sufficient to substantially reduce neutralization of sera at the NT_100_ dilution. Increasing the amounts of pentamer and trimer in these experiments did not substantially increase depletion of trimer- and pentamer-specific NAbs. The pentamer and trimer are considered to be in native conformations because they bind to cell surfaces, block entry of HCMV into cells, and were recognized by conformational-specific MAbs (34). All three proteins contained poly-His tags on gH which were used to remove the soluble proteins and attached Abs using nickel agarose. The antibody-depleted sera were then characterized for neutralization by incubation with HCMV BAD*r*UL131, a virus expressing the pentamer and GFP (35), before infection of ARPE-19 epithelial cells. A human serum from a seronegative donor was used as a negative control (ctl serum). Depletion with soluble pentamer substantially reduced neutralization with all of the sera by 50–90% (Fig. 1 and *SI Appendix*, Fig. S1). The majority of these sera also showed significant depletion of NAbs following incubation with the soluble trimer, although, frequently, depletion with the trimer reduced NAbs less than depletion with the pentamer. There were more limited examples in which the soluble trimer was as effective or more effective in depleting NAbs compared with the pentamer (Fig. 1, sera 117 and 025, and *SI Appendix*, Fig. S1, sera 051 and 290). Two sera did not appear to contain trimer-specific NAbs (Fig. 1, sera 004, and *SI Appendix*, Fig. S1, sera 179). Depletions with soluble gH/gL did not reduce neutralization with any of the sera tested (Fig. 1 and *SI Appendix*, Fig. S1). We also tested a subset of transplant sera after depletion for neutralization of HCMV on endothelial cells (HUVECs). Like for epithelial cells, depletion assays showed trimer- and pentamer-specific antibodies in transplant sera 190, 227, 158, 117, and 39 that were able to neutralize infection of HCMV into HUVECs (*SI Appendix*, Fig. S2). In addition, we performed depletion and neutralization assays with a second HCMV, strain TR, that produced results similar to those of BAD*r*UL131 on epithelial cells (*SI Appendix*, Fig. S3*A*). We could not characterize the Abs that bound to the trimer and pentamer because this would involve eluting the Abs and also the trimer and pentamer proteins that block HCMV infection of cells (34). We concluded that the majority of these transplant sera contain NAbs that recognize both the trimer and pentamer.

### Trimer-Specific NAbs Increase over Time.

**Fig. 2.**
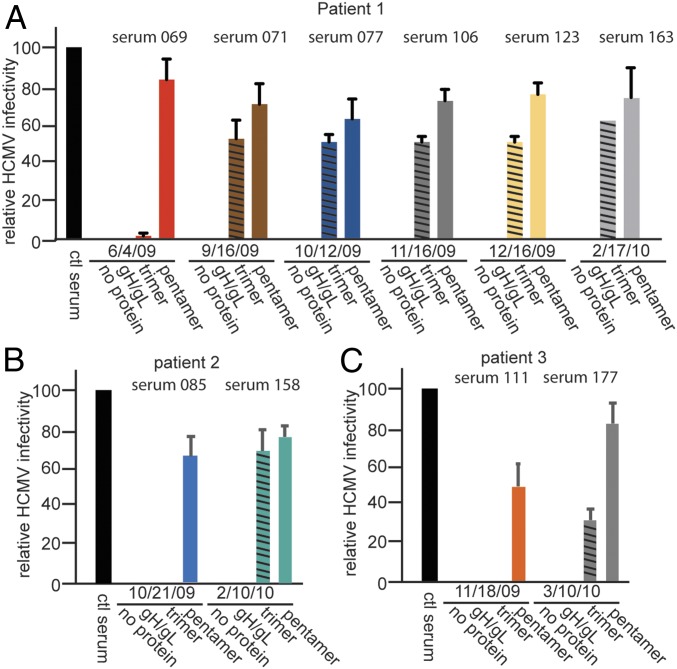
(*A*–*C*) Progression of NAbs over time in human transplant patients. Sera from three transplant patients (patient 1, patient 2, and patient 3) not shown in Fig. 1 were collected at various times following transplantation (with the indicated dates). The sera were diluted to NT_100_, then depleted of NAbs with gH/gL, trimer, or pentamer as in Fig. 1. The depleted sera were then tested for neutralization of HCMV on ARPE-19 cells.

To determine whether NAbs changed over the course of time, we characterized sera drawn over a period of 4–8 mo from three additional transplant patients not shown in Fig. 1. Again, we depleted gH/gL-, trimer-, and pentamer-specific Abs and measured neutralization. In one of these transplant patients (patient 1, sera 069, 071, 077, 106, 123, 163), NAbs were characterized over an 8-mo period following transplantation (Fig. 2*A*). In the first blood draw, few trimer-specific NAbs were detected; there were only 3–5% reductions in NAbs following depletion with the trimer. However, trimer-specific NAbs increased in the next 3 mo (blood draw September 16, 2009) and remained relatively stable over five additional months (Fig. 2*A*). Blood drawn from a second transplant patient (patient 2) initially showed no trimer-specific NAbs, but after 4 mo there were substantial trimer-specific NAbs (Fig. 2*B*). A similar profile was observed with a third transplant patient (patient 3), with no trimer-specific NAbs detected following depletion with the trimer in the earliest sample followed by significant trimer-specific NAbs observed 4 mo later (Fig. 2*C*). Strong pentamer-specific NAbs were observed at all times sera were collected, while no depletion of NAbs occurred with gH/gL at any time (Fig. 2).

### Both Trimer- and Pentamer-Specific NAbs Were Observed in Sera from Pregnant Women, and These NAbs Did Not Correlate with HCMV Transmission to the Fetus.

**Fig. 3.**
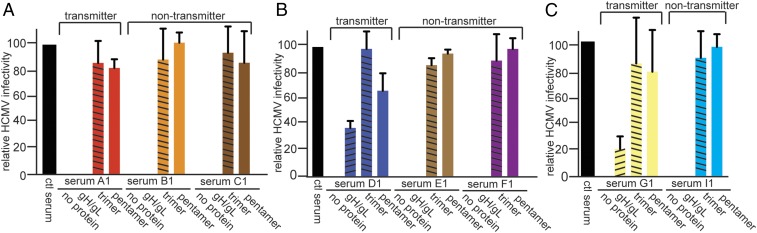
(*A*–*C*) Effects of depleting trimer- and pentamer-specific Abs from sera from pregnant mothers. Sera were compared based on mothers with similar ages and demographics, and each group is shown in separate panels that included one mother who transmitted HCMV to her baby and one or two mothers who did not transmit the virus. All of the sera shown here were collected during the first trimester. Sera were diluted to NT_100_ values, then depleted using no protein or 1 μg of gH/gL, trimer, or pentamer. Neutralization of HCMV was determined using ARPE-19 epithelial cells. There were 14 individual mother donors, designated A through O. Data obtained with sera from donors A–I are represented here, and data obtained with sera from donors J–O are represented in *SI Appendix*, Fig. S4 *A* and *B*. Note that serum from donor H was not included due to poor neutralizing activity.

Sera were collected from a cohort of 14 pregnant Brazilian women (donors A through O) who are part of a population with very high rates (>97%) of HCMV seropositivity (12). Previous studies in this population have shown that most women are infected at a young age (11). Sera were obtained during the first, second, and third trimesters. These patients were divided into groups of two or three women based on similar ages and demographics. Each group included one woman who transmitted HCMV to the baby (transmitter) and one or two women who did not transmit HCMV (nontransmitters) (each group is shown in a separate panel in Fig. 3 and *SI Appendix*, Fig. S4). Diagnosis of congenital infection of infants after birth was made by detection of HCMV by PCR analyses of saliva and urine. Neutralization titers were determined using ARPE-19 cells (*SI Appendix*, Table S2). NT_100_ dilutions were used in depletion studies involving gH/gL, the trimer, and the pentamer as described above. Sera drawn from these patients in the first trimester were denoted with the numeral 1 (e.g., A1, B1, etc.), second-trimester sera were denoted with the numeral 2 (e.g., A2, B2, etc.), and third trimester sera were denoted with the numeral 3 (e.g., A3, B3, etc.). First-trimester sera showed evidence of both trimer- and pentamer-specific NAbs, and in all cases, the efficiency of depletion involving the trimer was similar to depletion with the pentamer (Fig. 3 and *SI Appendix*, Fig. S4 *A* and *B*). Similar results were found with HCMV TR (*SI Appendix*, Fig. S3*B*). With a limited number of these maternal sera, we also observed depletion of NAbs by gH/gL, although there was less depletion of NAbs with gH/gL compared with depletions involving the trimer and pentamer in every case (Fig. 3 *B* and *C*, donor sera D1 and G1, and *SI Appendix*, Fig. S4 *A* and *B*, donor sera L1 and N1). We also compared NAbs in sera from six donors (A–F) for changes in these NAbs during the second and third trimesters (*SI Appendix*, Fig. S5). Again, there were similar depletions with both the trimer and pentamer of NAbs in all sera from both the second and third trimesters.

Importantly, no major differences in trimer- and pentamer-specific Abs were observed when comparing women who transmitted HCMV to babies with women who did not transmit the virus. For example, donors A1, D1, and G1 had high titers of NAbs specific for the trimer and pentamer but transmitted HCMV to babies, while patients B1, C1, E1, F1, and I1 did not transmit HCMV and had similar titers of NAbs specific for the trimer and pentamer (Fig. 3). Similar results were seen for transmitting patients J1 and M1 compared with matched nontransmitting patients K1, L1, N1, and O1 (*SI Appendix*, Fig. S4 *A* and *B*). Mann–Whitney statistical analyses of these data showed that there was not a significant difference in pentamer- and trimer-specific NAbs in transmitters versus nontransmitters (*SI Appendix*, Fig. S6). In contrast to a previous report, we concluded that trimer- and pentamer-specific NAbs do not predict transmission of HCMV to babies in this population of women (5).

### Trimer- and Pentamer-Specific Abs Act Synergistically for Efficient Neutralization of HCMV Infection of Epithelial Cells.

**Fig. 4.**
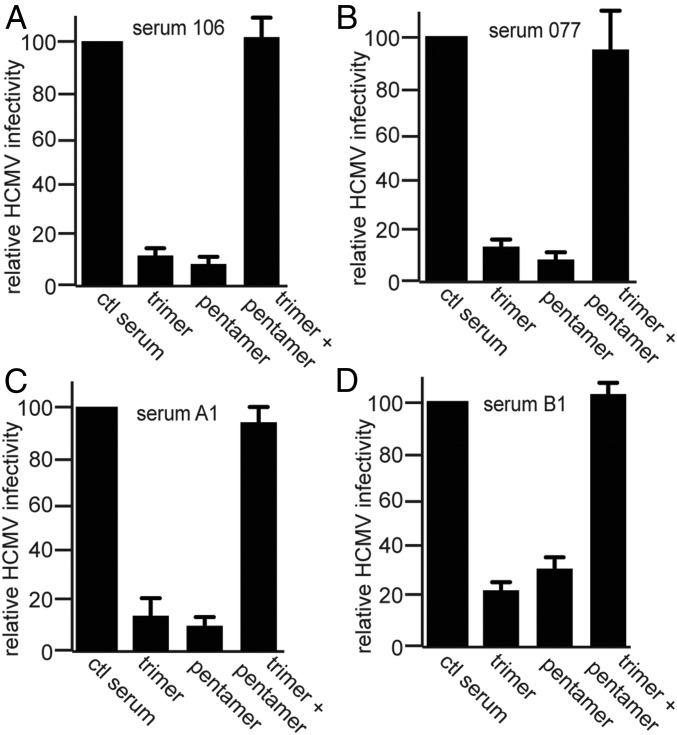
Synergistic effects of trimer- and pentamer-specific NAbs on HCMV neutralization. Sera from either transplant patients, sera 106 and 077 (*A* and *B*), or pregnant mothers, sera A1 and B1 (*C* and *D*), were diluted to NT_100_ values and then depleted using the soluble trimer or pentamer or depleted with both the soluble trimer and pentamer. In contrast to Figs. 1–3, depletions were performed with 0.125 μg of each of the soluble proteins to reduce the efficiency of the single-protein depletions to measure differences in the codepletions. The depleted sera were then mixed with HCMV and infectivity tested using APRE-19 cells.

Both trimer and pentamer depletion markedly depleted NAbs in most of the human sera from both transplant patients and mothers. Frequently, the depletion was nearly complete with either the trimer or pentamer. For example, with sera A1 (Fig. 3*A*), the trimer reduced neutralization by 85%, and the pentamer reduced neutralization by 82%. At first these results might appear surprising if one assumes that these effects should be additive. However, these results suggested that the most efficient neutralization of HCMV infection of epithelial and endothelial cells with human sera requires both trimer- and pentamer-specific Abs. In other words, depletion of either trimer- or pentamer-specific NAbs limits the capacity of the polyclonal Abs to effectively neutralize HCMV at the NT_100_ dilution. To address this, we performed depletion assays with NT_100_ diluted sera (two transplant sera and two mothers’ sera) using lower quantities of soluble trimer or pentamer complexes (0.125 μg compared with 1 μg in Figs. 1–3). Depletion with 0.125 μg of the trimer or 0.125 μg of the pentamer reduced neutralization by only 8–31% (Fig. 4), substantially less than observed in Figs. 1–3 with 1 μg of either protein. However, when these sera were depleted by incubation with both 0.125 μg of the trimer and 0.125 μg of the pentamer, virus neutralization was reduced by 92–100% (Fig. 4, trimer + pentamer). We concluded that the trimer- and pentamer-specific NAbs synergize in neutralizing HCMV; that is, there were more than additive effects.

### Trimer- and Pentamer-Specific Abs Prevent Virus Spread Within Epithelial Cells.

**Fig. 5.**
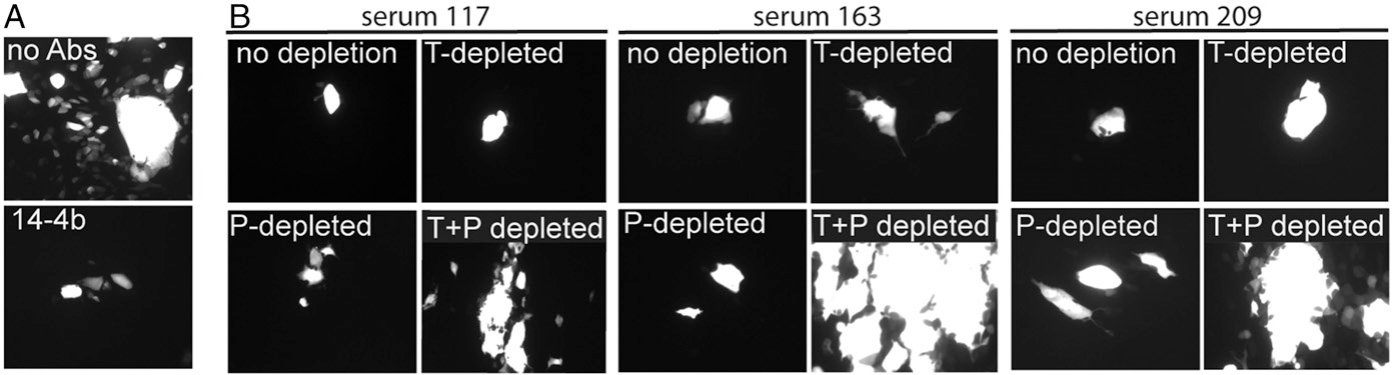
Trimer- and pentamer-specific Abs act together to reduce HCMV cell-to-cell spread. (*A*) ARPE-19 cells were infected with HCMV (BAD*r*UL131) that expresses GFP, then trypsinized and plated with uninfected ARPE-19 cells in the presence of no Abs or with anti-gH MAb 14-4b. (*B*) Similarly, cells were incubated with sera from three different transplant patients (sera 117, 163, and 209) that had been depleted of Abs by incubation with no protein (no depletion) or with 1 μg of the trimer (T) or pentamer (P) or with both the trimer and pentamer (T+P). The virus was allowed to spread for 10 d, and GFP expression in cells was characterized by fluorescence microscopy. Images were acquired with 100× magnification.

The effects of NAbs described above involved neutralization of infectious cell-free virus particles. HCMV and other herpes viruses spread directly from cell to cell, and Abs can dramatically curtail HCMV spread between ARPE-19 epithelial cells. MAb 14-4b, specific for gH, recognizes both the trimer and pentamer, neutralizes HCMV (36, 37), and substantially blocks the spread of HCMV BAD*r*UL131 in ARPE-19 cell monolayers (Fig. 5*A*). Transplant patient sera 117, 163, and 209 also dramatically reduced the spread of HCMV (Fig. 5*B*, no depletion). Depletion of these sera with either the trimer or pentamer did not substantially reduce the ability of these sera to prevent virus spread (Fig. 5*B*). By contrast, depletion of these sera with both the trimer and pentamer together substantially reduced the inhibitory effects of the sera in reducing virus spread (Fig. 5*B*). We concluded that trimer and pentamer NAbs act synergistically to reduce HCMV spread between ARPE-19 cells.

## Discussion

There is intense interest in the HCMV pentamer as an antigen that evokes strong antibody responses and as a potential component of subunit and virus-based vaccines. However, there have been no studies comparing pentamer-specific Abs with Abs specific to the other major form of gH/gL in virions, the gH/gL/gO trimer, which is essential for entry of extracellular virus into all cell types (23). We assessed whether there were trimer-specific NAbs in sera from human heart transplant patients by depleting specific NAbs by incubation with soluble forms of gH/gL, the trimer, and the pentamer (34). In all but a few cases the transplant sera displayed substantial loss of neutralization following incubation with the trimer. In many cases, the pentamer was somewhat more effective in diminishing NAbs compared with the trimer, although there were also examples of equal or higher levels of NAb depletion with the trimer. There was also evidence that pentamer-specific NAbs might arise earlier than trimer-specific NAbs. None of the transplant sera showed diminished NAbs when incubated with gH/gL. Note that two of the transplant patients (025 and 110) received Cytogam, which contains human Ig. Given that this human Ig was from HCMV-infected individuals, it does not alter our conclusion below. We observed analogous results with sera from pregnant mothers; that is, depletion with the trimer reduced NAbs similarly to pentamer depletion. With these sera there was limited evidence of NAbs that were depleted with gH/gL. We concluded that human sera contain NAbs specific for both the trimer and pentamer, but few showed functionally important gH/gL-specific NAbs.

There was also evidence that trimer- and pentamer-specific Abs could synergize in blocking HCMV infection of cells. When sera were incubated with lower concentrations of the trimer or pentamer (0.125 μg instead of 1 μg), the neutralization capacity of the sera was largely reduced (8–31%). However, when the trimer and pentamer were combined at these lower concentrations, there were 92–100% reductions in neutralization. One explanation of these results suggests that depletion with 0.125 μg of the pentamer removes certain pentamer-specific NAbs but leaves behind other pentamer-specific NAbs and all of the trimer-specific NAbs. However, combined depletion with 0.125 μg of both the trimer and pentamer removes sufficient quantities and specificities of both trimer- and pentamer-specific NAbs so that neutralization is abolished. These results demonstrated more than additive effects and thus were evidence of synergistic effects of these Abs, which are recognizing two distinct proteins in the virion envelope. Recently, we reported that the soluble trimer blocks HCMV binding to epithelial and endothelial cells, while the pentamer does not block virus binding but apparently acts downstream of the trimer to block a subsequent stage of virus entry, resulting in the exit of the virus from endosomes into the cytoplasm (34). Thus, trimer-specific Abs might block binding to cells, while pentamer-specific Abs exacerbate virus delivery into endosomes or membrane fusion delivering the virus to the cytoplasm. Trimer- and pentamer-specific Abs in human sera also acted in a synergistic fashion to reduce HCMV spread between epithelial cells, an important process in vivo.

Given the substantial reductions in NAbs with both the trimer and pentamer, one might expect that many of the Abs acting in neutralization would recognize gH/gL, which represents the scaffold that gO or UL128-UL131 assembles onto. However, for the most part, this did not appear to be the case, which was surprising. Mouse gH/gL-specific MAbs can effectively neutralize HCMV (38), but perhaps these Abs are not generated as efficiently during infection compared with immunization that occurs during virus infection. Alternatively, many gH/gL-specific Abs in sera may not effectively recognize epitopes in the pentamer and trimer because these epitopes are masked by UL128, UL130, UL131, and gO.

Our studies with sera of pregnant mothers showed no differences in the titers of trimer- and pentamer-specific NAbs when comparing mothers who transmitted HCMV with mothers who did not transmit the virus. There were also no real differences in the total NAb titers in sera when comparing transmitters with nontransmitters (*SI Appendix*, Table S2). Thus, we concluded that trimer- and pentamer-specific NAbs do not correlate with transmission from mothers to babies, a conclusion that differed from that reported by Lilleri et al. (5). Women in their studies underwent primary infection shortly before or during pregnancy (primary infection). It has been argued that primary infection carries a higher risk for transmission to babies (39). However, there has been debate about whether there is increased risk of transmission in these women (primary infection) compared with women who acquire the virus long before pregnancy (nonprimary infection) (4). In our studies, the sera from mothers were derived from Brazilian women who were >95% seropositive and carried high concentrations of NAbs likely from childhood. However, given the high prevalence of HCMV in this maternal population, reinfections with new strains of HCMV during pregnancy is likely frequent (40). Reinfection during pregnancy might lead to more active virus replication and intrauterine transmission. Some estimates have suggested that the number of congenitally infected infants in pregnancies following primary infection is significantly less than the number of congenitally infected infants born after nonprimary infection (4). It was reported that about 70–80% of congenitally infected infants are born to women undergoing nonprimary infection during pregnancy (41). In addition, the maternal specimens utilized in this study were derived from a population in which nearly 90% of infants with congenital HCMV infection were born to women undergoing nonprimary infections during pregnancy (12). Given these considerations, we would expect that our observations that trimer- and pentamer-specific NAbs do not determine outcome apply to a much larger population of congenitally infected infants from mothers who acquire HCMV while pregnant. Further, given the strongly neutralizing capacities of both trimer- and pentamer-specific NAbs, our findings provide additional evidence supporting the conclusion that maternal immunity in the form of Abs provides incomplete protection from congenital HCMV infection (4). That said, we have not yet measured whether other effects of these Abs, for example, involving complement or antibody-dependent cell-mediated cytotoxicity, are protective.

## Materials and Methods

### Soluble Proteins.

Soluble gH/gL, gH/gL/gO trimer, and gH/gL/UL128/UL130/UL131 pentamer complexes were produced by plasmid-expressing proteins in 293 6E cells followed by affinity chromatography purification from tissue culture supernatants as previously described (34).

### Human Sera.

Human sera from heart transplant patients were obtained at Oregon Health & Science University (OHSU) with patient consent. All experiments involving human subjects were approved by our OHSU IRB Protocol 0004474. Heart transplant patient sera were obtained during routine monitoring. Heart donor and recipient status, antiviral treatment, and the history of detectable viremia are provided in *SI Appendix*, Table S1. Maternal sera were derived from women enrolled in a prospective study of HCMV infection in a highly seropositive population in São Paulo, Brazil. Women were enrolled at their first antenatal visit with follow-up evaluations in the second (20–26 wk) and third (32–36 wk) trimesters of gestation and at 1 mo after delivery. Maternal first trimester sera were obtained at a median gestational age of 8 wk. Infants born to women enrolled in this study were screened for HCMV infection by testing the saliva obtained within 1 wk of age for HCMV DNA using PCR and by confirming the diagnosis of congenital CMV by testing urine samples from these infants within 3 wk of age by PCR (12). All study procedures were approved by the local and National Committee for Ethics in Research (16.928/2013), and written informed consent was obtained from the subjects.

### Serum Depletion Assay.

Sera diluted to NT_100_ titers in Opti-MEM without FBS (50 μL) were incubated with 1 μg (or 0.125 μg for the experiments described in Fig. 4) of soluble gH/gL, trimer, or pentamer proteins, which contain poly-His domains (34), for 1 h at 37 °C; then 15 μL of nickel nitrilotriacetic acid agarose (Invitrogen) were added and incubated at 23 °C for 1 h with agitation. The sera were centrifuged in affinity purification spin columns (Pierce) at 1,000 × *g* for 30 s to separate the sera from gH/gL soluble proteins bound to nickel agarose. The depleted sera and control sera were then tested for neutralization of HCMV BAD*r*UL131.

## Supplementary Material

Supplementary File
