# Supplementary material for: HCMV trimer- and pentamer-specific antibodies synergize for virus neutralization but do not correlate with congenital transmission
Source: Proc Natl Acad Sci U S A. 2019 Feb 7;116(9):3728–33. doi: 10.1073/pnas.1814835116 (PMC6397592; doi:10.1073/pnas.1814835116)
Supplement: Supplementary File [file pnas.1814835116.sapp.pdf]

PNAS
www.pnas.org

Supplementary Information for

HCMV trimer- and pentamer-specific antibodies synergize for virus neutralization but do not correlate with congenital transmission.

Adam L. Vanarsdall[a], Andrea L. Chin[a], Jing Liu[b], Theodore S. Jardetzky[b], James O. Mudd[c], Susan L. Orloff[d], Daniel Streblow[e], Marisa M. Mussi-Pinhata[f], Aparecida Y. Yamamoto[f], Geraldo Duarte[g], William J. Britt[h] and David C. Johnson[a1]

Corresponding author: David C. Johnson
Email: johnsoda@ohsu.edu

This PDF file includes:

Supplementary text
Figs. S1 to S5
Tables S1 to S2
References for SI reference citations

www.pnas.org/cgi/doi/10.1073/pnas.1814835116

Supplementary information

SI Materials and Methods

Cells and viruses. Primary human neonatal dermal fibroblasts (NHDFs; Invitrogen) were grown in DMEM with 10% FBS. Human umbilical cord vascular endothelial cells (HUVECs) were a gift from Ashlee Moses (O.H.S.U) and were maintained in Medium-200 plus low serum growth supplement (Invitrogen). Human retinal pigmented epithelial (ARPE-19) cells were obtained from ATCC and grown in DMEM/F12 plus 10% FBS. HCMV BAD*r*UL131 from Tom Shenk (Princeton University) is a derivative of AD169 with UL131 repaired and encodes a GFP reporter gene (1). HCMV strain TR used in our lab was described (2). HCMV stocks were grown on NHDFs and tittered as described (3).

Virus neutralization assays. Sera were treated at 55°C for 30 min then diluted 1:10 followed by a 2-fold dilution series in a volume of 50 μl of Opti-MEM media in order to calculate neutralization titers. The diluted sera werer mixed with HCMV BAD*r*UL131 virus particles sufficient to infect 25% of ARPE-19 cells or 20% of HUVECs in 96 cell dishes and the virus and sera incubated at 37°C for 1 hr. The virus and sera were then added to cells in 96-well dishes for 2 hr then 50 μl of growth media was added to the wells and the cells incubated for 24 hr. Virus infectivity was assessed by monitoring GFP expression, comparing the number of GFP positive cells to the total number of cells. For infectivity of HCMV TR we infected ARPE-19 cells for 24 hr then stained the cells with IE-86 Abs (2). Serum dilutions that produced 50% reductions in virus infectivity (NT_{50}) or 100% reductions (NT_{100}) were determined.

Cell-cell spread assay. ARPE-19 cells were infected with HCMV BAD*r*UL131 for 24 hr then the cells trypsinized and mixed with uninfected ARPE-19 cells at a ratio of 1:1000 then this cell mixture seeded into 96-well culture dishes with growth media. After 24 hr depleted human sera depleted with 1 μg of trimer or pentamer or a control sera from a seronegative patient or anti-gH MAb 14-4b (1 μg/ml) were added to the cell monolayers for 10 days at 37°C. Fresh media containing sera or Abs were replenished every 2 days.

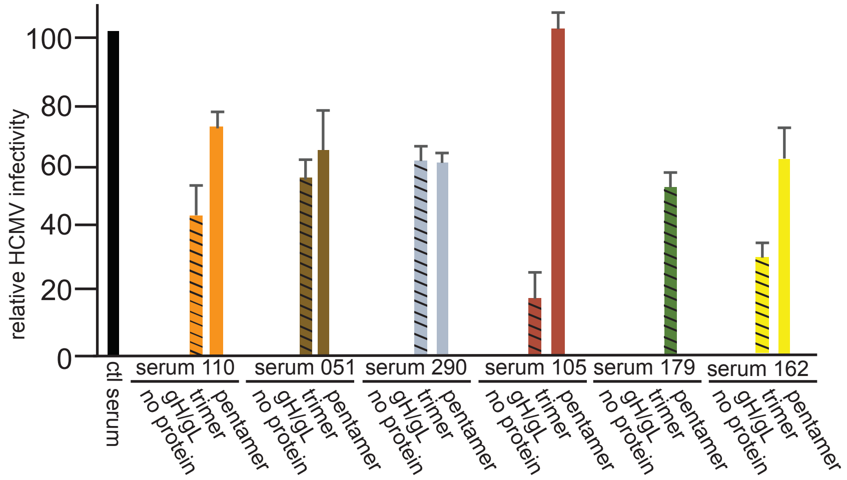

Fig. S1. Depletion of neutralizing antibodies from human transplant patients. Human sera collected following heart transplant were diluted to titers that resulted in 100% neutralization of HCMV infection of APRE-19 cells. The diluted sera were then incubated with either no protein or with soluble gH/gL, trimer, or pentamer and then tested for the ability to neutralize HCMV (BAD*r*UL131) infection of APRE-19 epithelial cells as describd for Fig. 1. The relative infectivity of HCMV after incubation with sera was determined by counting GFP+ (infected) cells after 24 h of infection and compared to the number of infected cells following incubation of HCMV with a seronegative donor serum (ctl serum).

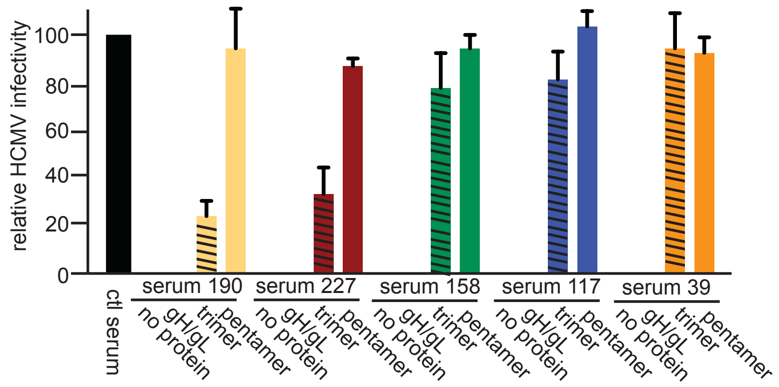

Fig. S2. Trimer and pentamer specific Abs from human transplant patients neutralize HCMV infection of endothelial cells. Sera from transplant patients were diluted to $NT_{(100)}$ values then depleted as described in Fig. 1. The depleted sera were incubated with HCMV (BAD*r*UL131) and infectivity was tested using human umbilical vein endothelial cells (HUVECs).

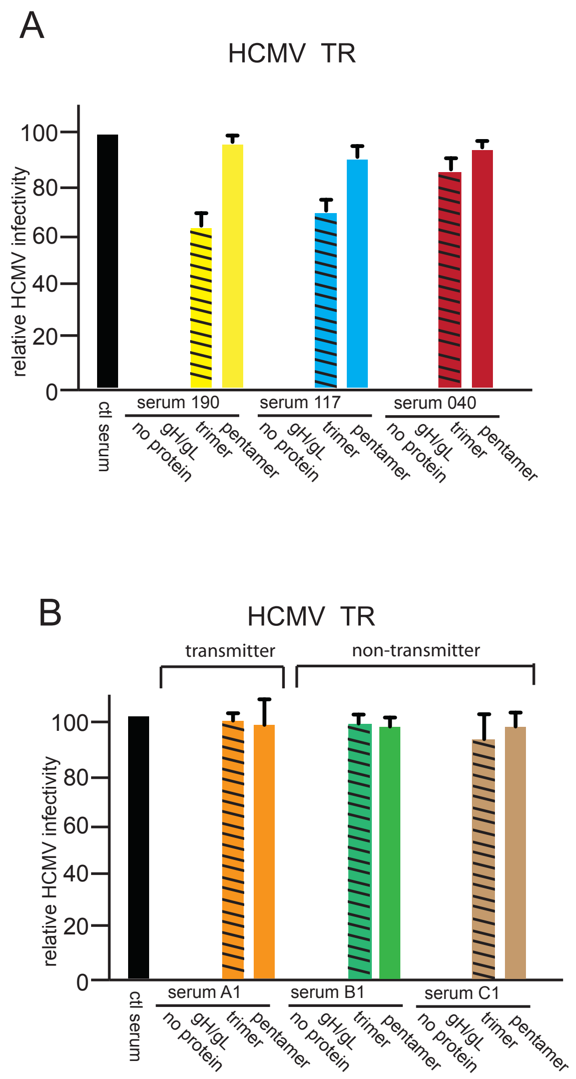

Fig. S3. Effects of depleting trimer- and pentamer-specific Abs on neutralization of HCMV strain TR. Three sera from transplant patients (panel A: sera 190, 117, and 040) and 3 sera from pregnant mothers (panel B: sera A1, B1,and C1) were diluted and subjected to antibody depletion as described for Figs. 1-3, then mixed with HCMV strain TR in neutralization assays. Virus was applied to ARPE-19 cells to test infectivity. After 24 hr the cells were fixed and stained with IE-86 Abs.

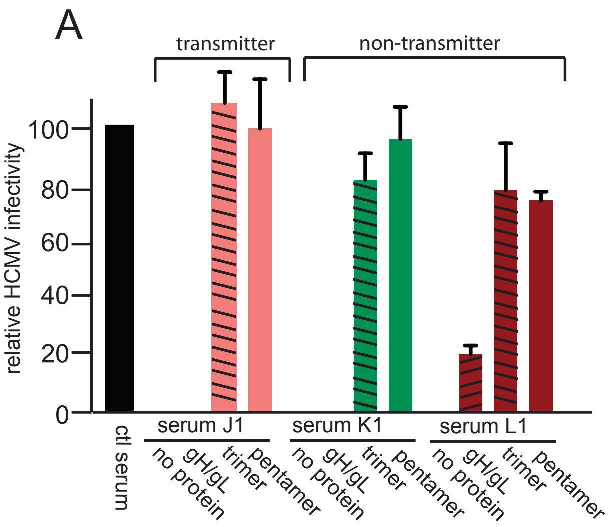

Fig. S4. Depletion of neutralizing antibodies from pregnant mothers sera. Panels A and B include sera from virus transmitting mothers compared to matched non-transmitting mothers with similar demographics. Sera were collected in the first trimester of pregnancy. The sera were subjected to antibody depletion with either no protein (as control), gH/gL, trimer, or pentamer as described for Fig. 3 and then used for virus neutralization assays with BAD*r*UI131 on ARPE-19 cells.

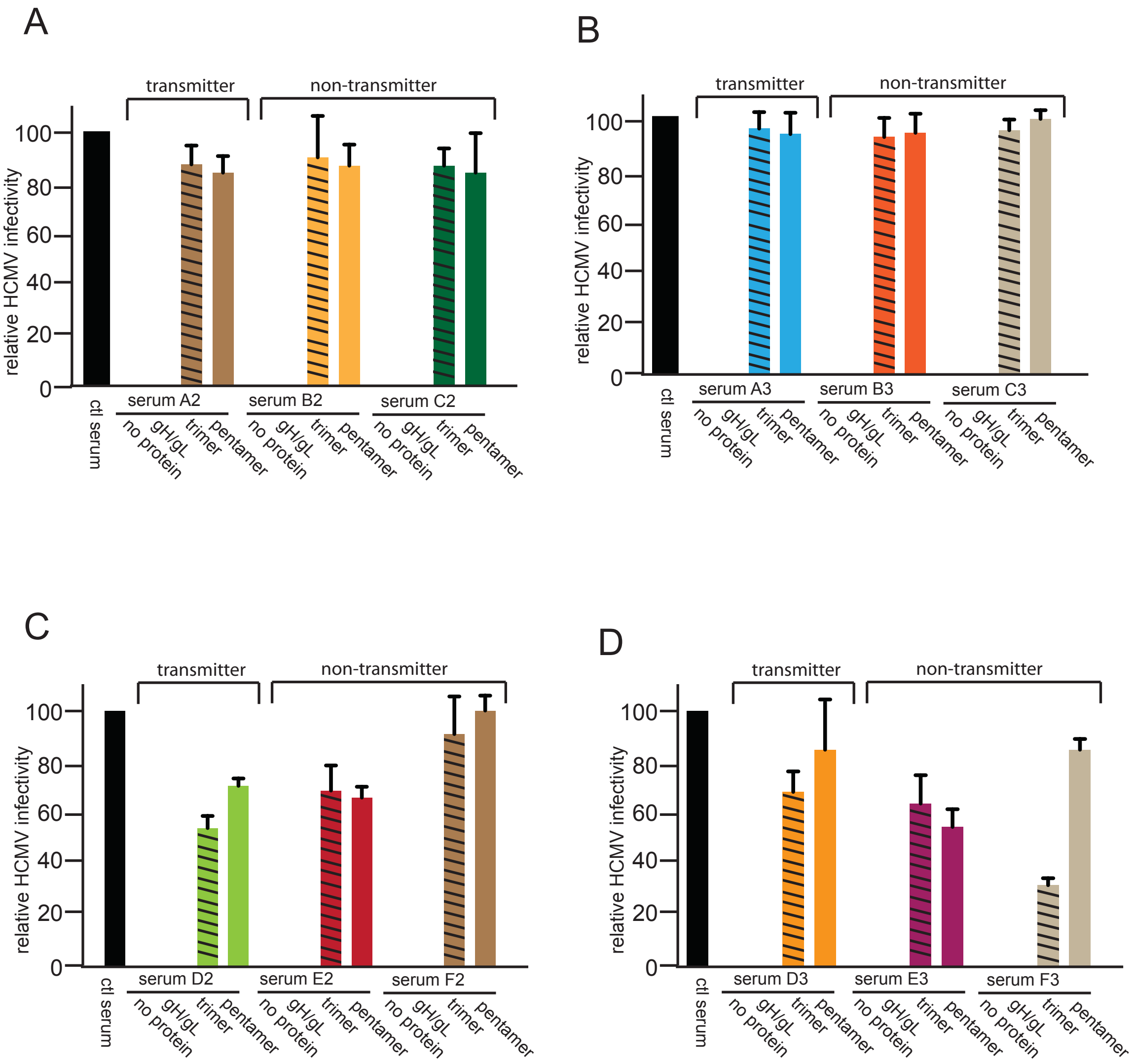

Fig. S5. Neutralization of HCMV by infected mothers sera collected during the second and third trimesters. Panels showing sera from virus transmitting mothers compared to matched non-transmitting mothers with similar demographics. Panel A includes sera from donors A, B and C collected during the second trimester and panel B includes sera from donors A, B and C collected during the third trimester. Panel C includes sera from donors D, E and F collected during the second trimester and panel D includes sera from donors D, E and F collected during the third trimester. Depletions were performed as in Fig. 3 using no protein, gH/gL, trimer, or pentamer.

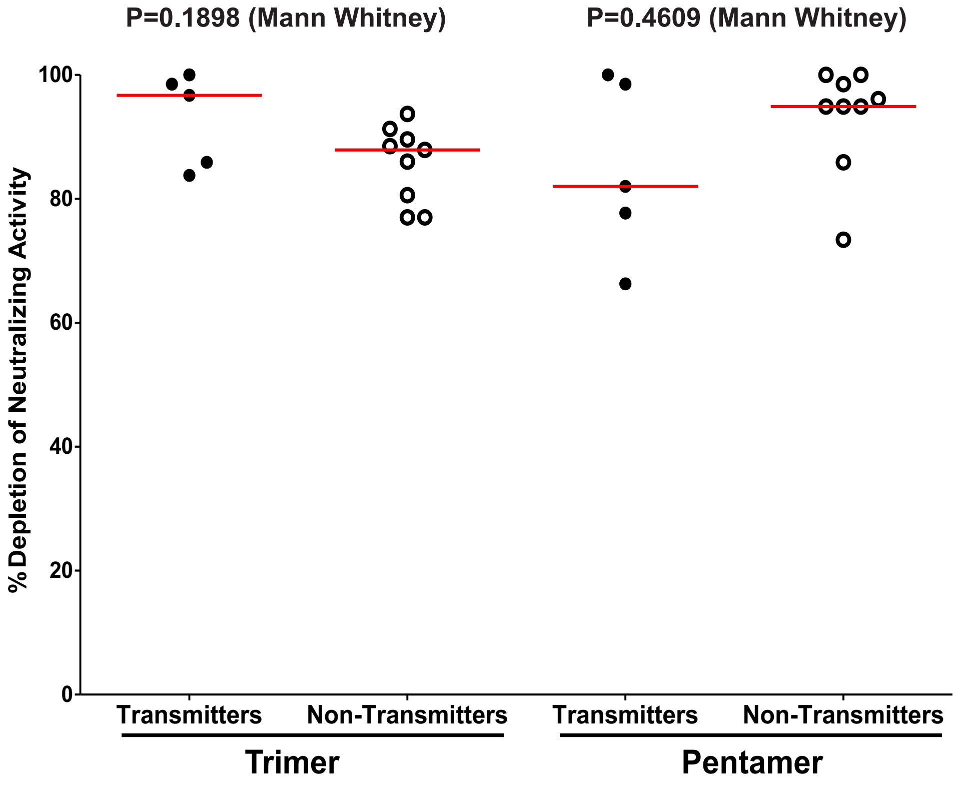

Fig. S6. Statistical analysis of the depletion of trimer- and pentamer-specific NAbs in mothers who either transmitted or did not transmit HCMV. Mann Whitney analyses of the pregnant mothers sera described in Fig. 3 and *SI Appendix, Fig*. S4 demonstrated that there was no significant difference in trimer- or pentamer-specific NAbs in sera derived from transmitters versus non-transmitters. P values are shown at the top of the figure.

Supplemental Table S1. Human Heart Transplant Patients

Serum Sample	Sex	D/R HCMV[a]	Antiviral Treatment	Viremia	Graft Failure	Notes
**004**	M	D+/R-	Valcyte	+		
**025**	M	D-/R+	Valcyte & Cytogam	+		
**085, 158**	F	D-/R+	Famvir			
**039**	F	D+/R+	Famvir		+	
**040, 105, 161**	M	D+/R+	Famvir			
**049**	M	D-/R-				
**051**	M	D+/R-	Valcyte	+		
**059**	M	D+/R+	Valcyte		+	
**069, 071, 077, 106, 123, 163**	M	D+/R+	Valcyte, Aciclovir, & IV Ganciclovir			
**110**	F	D+/R-	Valcyte & Cytogam			
**111, 177**	M	D+/R-	Valcyte & IV Ganciclovir	+	+	
**117**	F	D-/R+	Aciclovir		+	
**150**	M	D-/R+	Valcyte		+	
**179, 209**	M	D+/R+	Valcyte & Aciclovir		+	Transplanted twice due to chronic graft rejection
**162**	M	D+/R+	Aciclovir			
**190**	M	D+/R+	Valcyte			
**227**	M	D+/R-	Valcyte & IV Ganciclovir	+	+	Transplanted twice due to chronic graft rejection
**290**	F	D+/R+	Valcyte	+		

[a]Donor HCMV positive/negative (D+/D-), Recipient HCMV positive/negative (R+/R-).

Supplemental Table S2. **Neutralizing antibody titers for individual donor sera.**

Transplant patient donors

Donor serum	NT_{50} (ARPE-19 cells)	NT_{100} (ARPE-19 cells)
004	1:1280	1:320
025	1:320	1:160
027	1:2560	1:640
039[a]	1:2560	1:640
040	1:1280	1:320
049	1:2560	1:640
051	1:640	1:160
059	1:1280	1:320
069	1:320	1:160
071	1:320	1:160
077	1:320	1:160
085	1:640	1:160
105	1:640	1:160
106	1:320	1:160
110	1:1280	1:320
111	1:1280	1:320
117[a]	1:2560	1:640
123	1:320	1:160
150	1:160	1:40
158[a]	1:1280	1:640
161	1:1280	1:320
162	1:320	1:80
163	1:320	1:160
177	1:2560	1:640
179	1:2560	1:640
190[a]	1:2560	1:640
209	1:2560	1:640
227[a]	1:5120	1:1280
290	1:640	1:160

[a] The NT_{50} and NT_{100} titers for these sera were the same when tested on HUVECs.

Pregnant women donors

Donor serum[a]	NT_{50} (ARPE-19 cells)	NT_{100} (ARPE-19 cells)
A1	1:1280	1:640
A2	1:1280	1:640
A3	1:640	1:320
B1	1:1280	1:320
B2	1:640	1:320
B3	1:640	1:160
C1	1:640	1:320
C2	1:1280	1:320
C3	1:1280	1:640
D1	1:1280	1:640
D2	1:320	1:80
D3	1:640	1:320
E1	1:320	1:80
E2	1:160	1:80
E3	1:160	1:80
F1	1:80	1:40
F2	1:640	1:320
F3	1:320	1:80
G1	1:1280	1:640
I1	1:1280	1:320
J1	1:160	1:80
K1	1:1280	1:640
L1	1:640	1:320
M1	1:640	1:160
N1	1:620	1:320
O1	1:160	1:40

[a] Donor sera from 14 individual donors. The letters A-O refer to the individual donors and the numerals 1, 2, and 3 refer to the sera collected during the first, second, or third trimester of that individual donor.

REFERENCES

1. Wang D, Yu QC, Schroer J, Murphy E, Shenk T (2007) Human cytomegalovirus uses two distinct pathways to enter retinal pigmented epithelial cells. *Proc Natl Acad Sci U S A* 104(50):20037–20042.
2. Ryckman BJ, Jarvis MA, Drummond DD, Nelson JA, Johnson DC (2006) Human cytomegalovirus entry into epithelial and endothelial cells depends on genes UL128 to UL150 and occurs by endocytosis and low-pH fusion. *J Virol* 80(2):710–722.
3. Vanarsdall AL, Wisner TW, Lei H, Kazlauskas A, Johnson DC (2012) PDGF receptor-alpha does not promote HCMV entry into epithelial and endothelial cells but increased quantities stimulate entry by an abnormal pathway. *PLoS Pathog* 8(9):e1002905.
